# Ukraine's triple emergency: Food crisis amid conflicts and COVID‐19 pandemic

**DOI:** 10.1002/hsr2.862

**Published:** 2022-10-07

**Authors:** Goodluck Nchasi, Carolyn Mwasha, Moshi Moshi Shaban, Rose Rwegasira, Benardine Mallilah, Joshua Chesco, Anastasiia Volkova, Ashraf Mahmoud

**Affiliations:** ^1^ Department of Medicine Catholic University of Health and Allied Science Mwanza Tanzania; ^2^ Department of Dentistry, School of Dentistry Muhimbili University of Health and Allied Sciences Dar es Salaam Tanzania; ^3^ Department of orthopedics Mbeya Zonal Referral Hospital Mbeya Tanzania; ^4^ Department of Mechanical Engineering University of Dar es Salaam Dar es Salaam Tanzania; ^5^ Department of Medicine Kilimanjaro Christian Medical University College Kilimanjaro Tanzania; ^6^ Department of Medicine, Faculty of Medicine Lviv National Medical University Lviv Oblas Ukraine

**Keywords:** COVID‐19, food crisis, Russia–Ukraine conflicts

## Abstract

Globally, both Russia and Ukraine play a key role in food production. Both countries are known for their meticulous positions in producing and exporting wheat, maize, sunflower seed oil, and cotton seed oil. Although the conflict between Russia and Ukraine has been going on for more than 5 years, the recent invasion of Russia in Ukraine has endangered food security in Ukraine during the COVID‐19 pandemic. As COVID‐19 cripples the healthcare infrastructure of Ukraine, food insecurity challenges the civilian population to migration. As the conflict intensitifes, damages to properties, loss of lives, rise of infectious diseases, incremental rise in energy prices, and fuel consumption are some of the possible consequences. This commentary aims to highlight the different ways in which access to food has been endangered, the implications that food crisis has on the world, and thus provide key recommendations on what needs to be addressed to mitigate the rising risks of the food crisis in the world.

## 1 INTRODUCTION

Ukraine is the second‐largest country in Europe after Russia, with a total area of 600,000 km² and a population of 43.2 million people. Ukraine accounts for about 17% of global corn exports, 12% of wheat, and 30% of sunflower seeds, most turned into oil in Ukrainian factories.^1^ In addition, Ukraine and Russia together called the world's bread basket, are top producers and exporters of several important grains (such as wheat and maize) and vegetable oils. Together they average for 30% of the world's grain production and at least 50% of the world's sunflower seed oil and cotton seed oil.^2^ Even more, these countries' contribution to the production of agricultural incentives and fertilizers such as nitrogen and potash is significant. Russia and Ukraine have conflicted for more than 5 years but, on February 24, Russia invaded Ukraine causing chaos and exacerbating the already‐existing COVID‐19 pandemic. As of June 2022, the country had a total of 5,015,994 confirmed cases and 108,622 deaths, however, 31,455,954 vaccine doses have been administered as of February 26, 2022.^3^ In addition, the National Research Foundation of Ukraine reports a 30,000 predictive incidence of COVID‐19 in Ukraine per day.^4^ Moreover, the conflicts in Ukraine led to the loss of lives and substantial property damage including farms causing a shortage of food not only in Ukraine but globally. On June 21, 2022, the Office of the United Nations (UN) High Commissioner for Human Rights (OHCHR) reported 4662 civilian deaths, and a displacement of more than 3 million individuals although stating that the actual figure of displaced people could be higher.^5^

## 2 IMPLICATIONS

The Russian–Ukraine war has prompted workplace closures, and restrictions on production and trade thus having cascading effects on economic activity, food prices, and employment. In addition, Ukrainians suffer from challenges such as limited access to fresh water, poor sanitation, disruption of stable food supply, and re‐emergence of infectious diseases.^6^, ^7^ The presence of hostilities is one of the biggest stressors to Ukrainians thus subjecting them to serious mental health problems, including post traumatic stress disorder (PTSD), depression, and anxiety.^7^, ^8^ Fel et al. report about 37.3% of Ukrainians are diagnosed with PTSD, which is attributed by factors such as loss of a loved one, lack of health insurance, and displacement of residence where over 5 million Ukrainians have fled their homes to seek refuge in European countries such as Poland.^7^, ^9^, ^10^

The global food system is facing uncertainty as the Russia–Ukraine crisis puts one among the world's major breadbaskets in an imminent threat.^11^ The scarcity of food in some parts of Ukraine greatly affects the livelihood of Ukrainians.^6^, ^7^, ^10^, ^11^ Most of the energy for agricultural production including fuels, electricity, fertilizers, pesticides, and lubricants is reallocated into military use, thus rendering the people unable to produce enough food and thus subjecting people to malnuitrition.^1^, ^12^

The health system of Ukraine is functioning at reduced capacity due to conflicts, with millions of Ukrainians lacking essential drugs and treatments such as insulin and antihypertensives.^11^, ^13^ The majority of patients with kidney disease fail to get hemodialysis due to the inability of the patients and medical providers to reach the dialysis center during the rocket attacks, bombing, and the presence of active hostilities on the ground.^14^ In addition, about 4 million people including children and healthcare workers have fled the country thus leading rapid spread and outbreak of infectious diseases such as Polio and COVID‐19 due to disrupted surveillance chains,^9^, ^10^ where an estimated 5 million cases were reported in 2022 alone.^10^ Surprisingly, only 30% of the Ukraine population received at least one dose of a COVID‐19 vaccine.^10^ However, COVID‐19 boosters have been approved in adults above 18 years of age who have been at least 6 months past the date of the previous vaccination.^15^ In children, the impact of conflicts has caused major disruption to immunization programs including COVID‐19 in children above 5 years of age.^15^ Furthermore, severely ill COVID‐19 patients living in territories where hostilities are taking place do not have the opportunity to seek medical help because medical facilities have been bombed, and medical personnel are limited.^16^ Due to the conflicts going on between Russia and Ukraine, countries like Myanmar which have also been under a coup d'etat and COVID‐19 fail to import vaccines from Russia rendering them victims of the war within and conflicts of Ukraine and Russia.^17^

In the trade risk, there have been steep production and shipments of products causing shortages to occur and inflation of prices worldwide, accounting for the price risk.^18^, ^19^ There are also the exchange rates, debts, and economic growth risk, which is foreseen due to discrepancies of currencies that will take place. This will indeed affect investments in the country as well as their ability to import products to sustain their country's livelihood.^20^ This implies that with the agricultural and economic activities being down, hence causing prices to rise, the nation of Ukraine has an overall low purchasing power leading to a situation of food insecurity in the near future and malnutrition among the Ukrainians and those depending on it for food supply.^21^, ^22^

## 3 EFFORTS AND RECOMMENDATIONS

### 3.1 Food crisis in Ukraine

The World Food Program (WFP) started implementing the plan to assist with food insecurity in Ukraine. Considering restrictions to men on leaving the country, this leaves women and children to be the refugee population at risk of hunger.^23^ Following the official request from Ukraine, WFP has launched an operation aimed at providing food to the ones that flee from conflicts inside Ukraine. Priority actions intended at averting the food security crises include conducting a rapid food security assessment to better estimate needs; mobilizing food security assistance; providing hot meals in collective centers; distributing emergency food kits; and delivering agricultural inputs including vegetable garden seeds.^24^ However, challenges arise in the implementation of the program. To begin with, the fact that Ukraine has been one of the bigger global food suppliers and Ukraine's nearby countries depend on the wheat supply from Ukraine.^25^ The conflict has then put these countries at food insecurity that could otherwise help fleeing individuals. Second, there has been a global increase in acute food insecurity, that raised from 135 to 276 million since 2019 due to the COVID‐19 pandemic. The pandemic has been mentioned to be among the causes of food insecurity among western Ukrainians in 2021.^23^ It is our recommendation that, in the implementation of the WFP wonderful operation, paying attention to the global details on food insecurity is crucial.

### 3.2 COVID‐19 pandemic crisis

Ukraine struggled with mitigation measures of COVID‐19 even before the invasion by Russia. The challenge have been less vaccination coverage, this is due to less need to get immunized and insecurities toward the vaccine.^15^, ^26^ COVID‐19 cases in Ukraine have escalated since Russian's aggression in the country. By February 25, only 34.07% of Ukrainians were vaccinated, making it the least‐vaccinated country in Europe.^15^, ^27^ The 5th COVID‐19 wave in Ukraine due to omicron started in mid‐January 2021 expected to last up to April.^15^, ^28^ At the nearby end of February, weekly new infections reached 26,7391.^29^ The Ukrainian government has signed up the contract as an effort for more vaccination importation and consider children's vaccinations when approved. We recommend stakeholders increase vaccination rollouts to Ukrainians both in the country and in the refugee camps. We also recommend provision of hand sanitizers, and face masks to the affected communities by the crisis.

### 3.3 Conflict/war crisis

Russian invasion of Ukraine has been related to being the largest conflict in Europe since the Second World War. In times of war, the defining characteristics of an armed conflict are a total blockade of humanitarian aid, destruction of health facilities, and displacement of millions of people.^30^ The effect on the economy, health, society as well as all spheres of life.^23^, ^24^ Women and children are the vulnerable groups affected by the ongoing conflict in Ukraine. Armed forces have been raging in various countries like Tigray in Ethiopia and Gaza face dire healthcare consequences with prevalent instability, lack of adequate medical resources, and limited health‐related infrastructure. there has been struggling to respond to the pandemic.^30^, ^31^ We hereby recommend the two oppositions parts along world institution like UN to find an appropriate method to resolve the conflict or end them all altogether.

## 4 CONCLUSION

The Russian–Ukraine conflicts have caused havoc on food production and supply not only in Ukraine but also in the world. This has led to high prices of food all over the globe including in America. In addition, it has exacerbated hunger in most countries in the Middle East and Northern Africa. During this crisis, there has been a recent spike in COVID‐19 cases that can be attributed to the failure of adherence to COVID‐19 prevention measures including washing hands and wearing face masks due to a limited supply of water and masks, respectively. Furthermore, the world powers together with the UN should interfere and bring consensus to this conflict since it poses risk not only to the global food supply but also to peace and security. In other countries as well facing military warfare, it is important that decisive action is taken to restore democracy that has been lost for the effort to be present to tackle global health pandemics like the COVID‐19 crisis for in upholding this commitment the citizens are protected from warfare unrest and health danger.

## AUTHOR CONTRIBUTIONS

**Goodluck Nchasi**: Conceptualization; project administration; writing – review and editing. **Carolyn Mwasha**: Writing – original draft. **Moshi Moshi Shaban**: Writing – original draft. **Rose Rwegasira**: Writing – original draft. **Benardine Mallilah**: Writing – original draft. **Joshua Chesco**: Writing – original draft. **Anastasiia Volkova**: Writing – review and editing. **Ashraf Mahmoud**: Conceptualization; writing – review and editing. All authors have read and approved the final version of the manuscript.

## CONFLICT OF INTEREST

The authors declare no conflict of interest.

## TRANSPARENCY STATEMENT

The lead author Goodluck Nchasi affirms that this manuscript is an honest, accurate, and transparent account of the study being reported; that no important aspects of the study have been omitted; and that any discrepancies from the study as planned (and, if relevant, registered) have been explained.

## Data Availability

The authors confirm that no primary data was collected or associated with this article.
